# Effect of high-pressure homogenisation on the behaviour of oleosomes and milk fat globules at an air-water interface

**DOI:** 10.1016/j.lwt.2026.119285

**Published:** 2026-04-15

**Authors:** Amin Aliyari, Vincenzo di Bari, Liam P.D. Ratcliffe, Yuanzhang Dong, David Gray

**Affiliations:** a Food Materials Science Group, Division of Food, Nutrition and Dietetics, School of Biosciences, University of Nottingham, Sutton Bonington Campus, Loughborough, LE12 5RD, UK; b The Magnum Ice Cream Company (TMICC), Colworth Laboratory, Colworth House, Sharnbrook, Bedford, MK44 1LQ, UK

**Keywords:** Milk fat globule, Oleosome, Oil body, Homogenisation, Interfacial tension

## Abstract

Growing demand for healthy and sustainable foods is accelerating the replacement of dairy ingredients with plant-based alternatives. Oleosomes, also known as oil bodies, are natural lipid droplets from oilseeds that form stable emulsions and may function as plant-based substitutes for MFGs. Because stabilisation at the air-water interface is critical for aerated products such as ice cream and whipped cream, this study compares the surface adsorption behaviour of thermally treated lipid suspensions containing MFGs or oleosomes. Both systems were analysed in their native state (D4,3 = 4.12 ± 1.32 μm for MFGs and 2.08 ± 0.97 μm for oleosomes) and after high-pressure homogenisation using a Microfluidizer at 69, 138, and 207 MPa, applied for one or two passes. In 15 g/dL lipid-based suspensions, increasing homogenisation pressure and pass number significantly reduced MFG size. In contrast, oleosomes at the same concentration showed no significant diameter changes beyond 69 MPa. Treatment at 207 MPa for two passes substantially modified the MFG membrane, with caseins dominating newly formed globule interfaces. These compositional changes led to slower interfacial equilibration and higher surface tension relative to native globules. Overall, these findings clarify key interfacial differences between dairy and plant lipid globules relevant to ingredient substitution.

## Highlights

• Phase volume concentration affected fat droplet emulsification in the microfluidizer.
• Homogenisation intensity affected D_4,3_ in MFG, but not significantly in oleosomes.
• Shear forces altered fat globule membrane protein composition.
• Homogenisation increased surface tension and equilibrium time of emulsions.

## 1 Introduction

Cow's milk consists of fat (3.7%) in the form of milk fat globules (MFG), proteins (3.4%, 80% of which is casein, in the form of micelles, and 20% is whey), and carbohydrates/lactose (4.8%) (Mieth, 1975). When the milk fat is removed, plasma remains, and when casein is removed serum remains. MFGs are naturally occurring lipid droplets produced by mammary epithelial cells, serving as a source of energy and essential nutrients for the growth of newborns (Lopez, 2023). These globules are considered natural oil-in-water (O/W) emulsions, with a lipid core (primarily composed of triacylglycerols, TAGs) encased in a complex tri-layer membrane that represents 2–6% of the total MFG mass (Argov et al., 2008). MFGs structure is particularly complex due to their intricate membrane composition. It primarily consists of polar lipids, cholesterol, and proteins, along with molecules such as cerebrosides, enzymes, and glycoproteins (Lopez, 2023; Mieth, 1975; Singh, 2006). The composition of the milk fat globule membrane (MFGM) can vary significantly due to factors such as lactation period, environmental conditions, and diet (Couvreur et al., 2007). Additionally, MFGM is highly susceptible to damage during processing steps such as agitation and foaming. When damaged, surface-active components (such as caseins and whey proteins) can rapidly be adsorbed to the exposed areas, leading to compositional changes in the MFGM (Mieth, 1975; Singh, 2006).

Proteins are an integral part of MFGM, with a diverse protein profile that varies in molecular weight. Mucin I, xanthine oxidase, and butyrophilin are among the most abundant proteins within the MFGM structure (Singh, 2006).

Phospholipids are also key components of the MFGM structure. Approximately 65% of the total phospholipid content in whole milk is in the MFGM, while the remaining 35% exists as free phospholipids in the milk plasma. The phospholipid fatty acid profile includes predominantly long-chain fatty acids, with short-chain fatty acids being less common. Although phospholipid content varies, phosphatidylcholine, phosphatidylethanolamine, and sphingomyelin are the most abundant phospholipids in the MFGM (Singh, 2006).

The lipid phase within MFGs is not always entirely liquid; it can contain solid components depending on temperature and fatty acid composition. The proportion of solid fat in the lipid phase is referred to as the solid fat content (SFC), a crucial factor influencing droplet density and functional properties such as foamability and emulsification (Truong et al., 2014). The fatty acid profile of MFG lipids plays an important role in determining SFC at a given temperature. Variations in fatty acid composition originates from multiple factors, including environmental conditions, diet, and lactation stage (Djordjevic et al., 2019). These variations also influence the size and composition of MFGs, as the lipid profile is a determining factor for lipid droplet size. MFGs diameter typically ranges from 0.1 to 20.0 μm, with an average size of approximately 4.0 μm in bovine milk (Briard et al., 2003).

A similar lipid storage mechanism exists in plants, where lipid organelles serve as energy reservoirs for cellular metabolism (Tzen & Huang, 1992). Oleosomes (also known as oil bodies or lipid bodies) are natural plant-based lipid droplets primarily found in seed tissues (Nikiforidis, 2019). Like MFGs, oleosomes consist of a lipid core composed mainly of triacylglycerols. However, unlike MFGs, their lipid core is surrounded by a monolayer membrane. The composition of the oleosome membrane also varies by source but predominantly consists of phospholipids and specific proteins. The primary membrane protein, oleosin, is a hydrophobic protein with a molecular weight of 15–20 kDa. Oleosins play a crucial role in maintaining oleosome integrity and preventing flocculation and/or coalescence. In addition to oleosins, smaller amounts of caleosins and steroleosins are also membrane constituents (Abdullah et al., 2020; Nikiforidis, 2019; Tzen & Huang, 1992). Oleosomes size varies depending on the source and extraction conditions, with an average diameter ranging between 0.5 and 5.0 μm (De Chirico et al., 2018).

As previously discussed, the size of lipid droplets is a critical factor influencing their functional properties and potential applications. Consequently, controlling droplet size has been a focus of many studies (Juliano et al., 2011; Kiełczewska et al., 2021). Homogenisation is widely used to enhance emulsion stability by reducing droplet size of fat globules. Various homogenisation techniques have been reported, including high or ultra-high-pressure homogenisation (Hayes & Kelly, 2003; Thompson & Singh, 2006), ultrasonic homogenisation (Abesinghe et al., 2020), and combinations of different methods. In the dairy industry, standard homogenisation typically applies pressures ranging from 20 to 60 MPa (Dumay et al., 2013).

Microfluidization is a high-pressure homogenisation method involving shear forces to break down droplets. This technique offers several advantages, including rapid processing, continuous operation, and solvent-free emulsification (Thompson & Singh, 2006). A microfluidizer consists of an interaction chamber where the main flow is divided into two opposing streams that collide at high velocity, causing droplet breakage. If free surface-active material/emulsifiers are present in the system, they may adsorb onto the newly formed lipid droplets. This process may alter the composition of the adsorbed emulsifier layer compared to the native droplets before homogenisation. High-pressure shear-forced homogenisation techniques, such as microfluidization, can induce compositional changes in fat globules due to the intense forces applied, potentially modifying the adsorbed emulsifier layer and affecting the stability, density, and interfacial behaviour of lipid droplets (Cano-Ruiz & Richter, 1997; Lee & Sherbon, 2002).

Given the structural similarities between oleosomes and MFGs, oleosomes have been proposed as potential alternatives to MFGs in vegan food products (Nikiforidis, 2019). One key application of MFGs is in dairy products (such as ice cream manufacture). However, before incorporating oleosomes as a potential replacement for MFGs in such formulations, fundamental research is needed to assess their behaviour under different processing conditions.

Therefore, the primary objectives of this study were: **I.** To investigate the effects of high-pressure homogenisation on oleosome size, structure, and surface adsorption behaviour; and **II.** To directly compare these effects with those observed in MFGs under identical processing conditions.

By providing a comparison between oleosomes and MFGs during homogenisation, this study addresses a key knowledge gap and improves understanding of how this widely used processing technique influences the structural and interfacial properties of both lipid systems, which is essential for their application in aerated food products.

## 2 Materials and methods

### 2.1 Raw materials and ingredients

Dehulled fresh sunflower seeds (*Helianthus annuus*) were supplied by The Magnum Ice Cream Company (TMICC, Colworth Science Park, Sharnbrook, Bedford, UK). Sodium bicarbonate (purity >99.7%) and sodium hydroxide (purity 99.0%) were purchased from Honeywell Fluka (Germany). All other reagents were of analytical grade and were obtained from either Merck Millipore (Darmstadt, Germany) or Sigma-Aldrich (USA). MFG cream (non-homogenised and heat-treated), used as a source of native MFGs, was obtained from a local supplier (Nottinghamshire, UK). The MFG cream contained 50.2% (dry-weight basis) lipids and 8.1% (dry-weight basis) proteins and exhibited negligible lipase activity.

### 2.2 Fatty acid profile of sunflower oil and milk fat

#### 2.2.1 Preparation of the lipid phase

The fatty acid profiles of sunflower oil and milk fat were analysed following organic solvent extraction using a Soxhlet extraction unit (Gerhardt Soxtherm, SX PC 1.40, Gerhardt, Germany). Petroleum ether with 2% n-hexane was used as the extracting solvent (150 °C for 30 min), followed by using n-hexane to suspend the lipids. The sample preparation procedures for oleosomes and milk fat globules (MFGs) were as follows:

**Sunflower Oil Extraction:** Sunflower seeds were dried in a vacuum oven (Fistreem International Co. Ltd, Leicestershire, UK) at 40 °C for 48 h (seeds weight were checked in triplicate to ensure a constant mass was achieved). The dried seeds were then ground using a laboratory blender. The oil was subsequently extracted via Soxhlet extraction.

**Milk Fat Extraction:** MFG cream was freeze-dried using a freeze-dryer (Edwards Modulyo, UK) for five days. The dried cream was then subjected to Soxhlet extraction to obtain milk fat.

#### 2.2.2 Preparation of Fatty Acid Methyl Esters (FAME) and Gas Chromatography-Mass Spectrometry (GC-MS) analysis

To prepare the FAME, 700 μL of 10 mol/L KOH and 5.3 mL of methanol were added to each tube containing 200 μL of extracted lipid samples suspended in n-hexane (ratio of 1:100, mL: mL) followed by vortexing for 30 s to mix them together.

Tubes were incubated in a water bath pre-set at 55 °C for 90 min, with vortexing for 5 s at regular intervals. After incubation, samples were cooled in an ice bath for 10 min. Subsequently, 580 μL of 12 mol/L H_2_SO_4_ was added to each tube, followed by vortexing for 30 s. The tubes were then returned to the water bath for another 90 min with periodic mixing. After a second cooling step in an ice bath for 10 min, 3 mL of n-hexane was added to each tube, followed by mixing for 30 s and centrifugation at 2000×*g* for 10 min at 20 °C. The upper hexane-containing phase was carefully collected and stored at −20 °C for further analysis.

Fatty acid profiling was performed using gas chromatography-mass spectrometry (GC-MS) with a Thermo Trace 1300 gas chromatograph coupled to an ISQ 7000 mass spectrometric detector (Thermo Fisher Scientific, USA). The Supelco 37 Component FAME Mix was used as a standard and diluted with n-hexane at a known concentration. Both standards and samples were aliquoted into vials and placed in an autosampler.

The running conditions were as follows: Column: SP®-2560 Capillary GC; Run time: 40 min; Temperature profile: Initial temperature of 140 °C, ramped up to 240 °C (heating rate of 4 °C/min) and held throughout the measurements.

Data analysis was conducted using Chromeleon 7 software (Thermo Scientific), and results were exported as an excel file.

### 2.3 Recovery of oleosomes from sunflower seeds

Oleosomes were recovered from fresh dehulled sunflower seeds through a wet-milling process using a lab-scale juicer (Angel 8500s, Angel juicers, AM Naarden, the Netherlands) according to the method reported by Aliyari et al. (2025). To obtain oleosomes, firstly, sunflower seeds were soaked in sodium bicarbonate buffer (0.1 mol/L NaHCO_3_, pH 9.5, ratio of 1:4 g: g) for 1 h at 50 °C in a water bath. After soaking the buffer was drained, soaked seeds were inserted into the juicer. The initial product of the extruder was called as crude oleosomes or crude oil bodies (COBs). To purify the COBs (removing the exogenous proteins and fibre), COBs were diluted in sodium bicarbonate buffer (pH 9.5, ratio of 1:7 g:g) and centrifuged (Beckman Avanti JXN-30 Series, Beckman Coulter, USA) at 10000×*g* for 35 min at 4 °C. The top cream layer was collected carefully and called as washed oleosomes or washed oil bodies (WOBs). COB and WOB had 58.7% (dry-weight basis) and 81.2% (dry-weight basis) lipid and 18.0% (dry-weight basis) and 11.9% (dry-weight basis) proteins, respectively. To minimise the impact of lipase and microorganisms on oleosomes stability, oleosomes were thermally treated at 75 °C for 5 min according to the method previously described by Aliyari et al. (2025).

### 2.4 Homogenisation of milk fat globules and oleosome emulsions

Firstly, standardised similar concentration suspensions of oleosomes and MFGs (15 g/dL lipid-based) were made in ultra-pure water. Then, a microfluidizer (LM20, Microfluidics, USA) was used to homogenise the samples at different pressures (69, 138, and 207 MPa) with one and two passes (1x and 2x, respectively).

### 2.5 Particle size distribution measurements of milk fat globules and oleosome droplets and surface area

The distribution of lipid droplets in the emulsions were measured using a Particle Size Analyser (LS 13320, Beckman-Coulter, USA) based on the Mie theory of light scattering (Wriedt, 2012, pp. 53–71). Emulsions were added to the measurement chamber and the analysis started once the obscuration value reached to a suitable level. De Brouckere mean diameter (volume-weighed mean particle size, D4,3) was calculated using Equation (1):

Equation 1 $$D_{4,3}=\frac{\sum n.d^{4}}{\sum n.d^{3}}$$

Where n is the frequency of occurrence of droplets in a specific size class with a mean diameter of d.

Total cumulative surface area of MFG and oleosomes before and after homogenisation was estimated from the measured mean diameter. Droplets were assumed to be spherical. For each sample, the dispersed phase volume fraction was calculated from the lipid concentration (g/mL) after conversion to volume using the oil density. The interfacial area per unit volume of emulsion was then obtained using equation (2).

Equation 2 $$A=\frac{6\times \varphi }{D_{4,3}}$$

Where A is the total cumulative area per unit volume of emulsion and φ is the disperse phase volume fraction.

To enable comparison across emulsions of different concentrations, the interfacial area was also normalised to the volume of oil present, giving the surface area per unit volume of dispersed lipid phase.

### 2.6 Surface charge and pH value measurements of milk fat globules and oleosome droplets

Surface charge of the milk fat globules and oleosome droplets were measured by a Litesizer 500 (Anton Paar light sizer 500, Anton Paar GmbH, Graz, Austria) using the Omega cuvettes. Before the analysis, samples concentrations were fixed at 0.01 g/dL by diluting the emulsions in ultra-pure water. Analysis was carried out at 20 °C as reported in literature (De Chirico et al., 2020).

To measure the pH value of MFG and oleosome suspensions, a pH meter (Mettler Toledo FiveEasy Plus™, Switzerland) was used at room temperature. Prior to the measurements, the device was calibrated at the same temperature using ready-made pH buffer solutions (pH 9 followed by pH 4 solutions).

### 2.7 Bright field and fluorescence micrographs of milk fat globules and oleosome droplets

Micrographs of oleosome and milk fat globule emulsions were taken using the bright field microscopy technique by a light microscope (Nikon H600L, Japan). Diluted emulsions were placed on a microscope glass and covered with a microscope slide and then visualised at 40× magnification.

To see the state of fat droplets (intact or ruptured), droplets were also visualised using a fluorescence microscope (EVOS FL Auto, Thermo Fisher Scientific, USA). Prior to the visualisation, samples were stained with Nile red solution in water (0.001 g/dL). A 40× magnification was used to capture the images (Aliyari et al., 2025).

### 2.8 Lipase activity measurement in oleosome suspensions

Lipase activity of oleosome emulsions (control and homogenised) were measured according to the method reported by (Ruiz et al., 2004) with some modifications. Briefly, at first, an enzyme solution was made as the raw material for the enzymatic assay. Then, 20 mmol/L of the p-nitrophenyl laurate (p-NPL) in 2-propanol solution was prepared and sonicated for 3 min. Then, a 1:10 (mL:mL) dilution in sodium phosphate buffer-Triton X-100 was made until the formation of a clear solution. Then, 1 mL of the enzyme solution (prepared previously) was mixed with the same amount of the substrate mixture (1 mmol/L p-NPL, 5% 2-propanol, 0.6% Triton X-100, 50 mmol/L sodium phosphate buffer). The final mixture was incubated at 40 °C for 90 min. After cooling down the samples in ice, the absorbance of samples was measured using the spectrophotometry procedure (at 405 nm) and was compared with the blank sample (the reaction mixture without the enzyme solution). Prior to the experiments, a standard curve was built using the commercial lipase. Results were reported as enzymatic activity unit which is the activity of enzyme that can release 1 μmol of p-Nitrophenol (p-NP) and normalised by the amount of protein in 1 g of cream (dry basis).

### 2.9 Total aerobic plate count

Total aerobic microbial count was measured according to the method previously described by (Aliyari & Rezaei, 2021). Briefly, six series of dilutions were prepared using the stock sample (original sample). Then, samples were applied to the counting medium (plate count agar, PCA, which had been sterilised in an autoclave for 45 min at 121 °C) next to a Bunsen burner and were incubated for 48 h at 37 °C. Finally, the total number of colonies were counted, and total microbial content was calculated using Equation (3). Total aerobic colony count (ACC) was reported as colony forming units (CFU) per volume of sample (mL), where A is the number of colonies counted, D is the dilution factor, and V is the volume inoculated.

Equation 3 $$\text{ACC}\;\left(\frac{\text{CFU}}{\text{ml}}\right)=A\times \frac{1}{D}\times \frac{1}{V}$$

All the measurements were repeated 5 times.

### 2.10 Surface tension measurements

Surface tension of the OB and MFG emulsions were measured using the method reported by (Aliyari et al., 2025). A force tensiometer (Attension Sigma 700, Biolin Scientific Inc., Sweden) was used to measure the changes in surface tension of emulsions over time. The Du Nouy ring (made of platinum-iridium; radius: 9.58 mm, thickness: 0.1850 mm) was used as the measuring geometry. At first, 49 mL of ultra-pure water was added to the measuring cuvettes. After starting the analysis, 1 mL of O/W emulsions (with the initial concentration of 20 g/dL) were added gently around the edges of the cuvette so that the final concentration of the measuring emulsion was fixed at 0.1 g/dL. A 1 mL of ultra-pure water was added and used as the negative control to ensure surface disruption does not occur during the addition into the cuvette. Temperature of the measuring cell was adjusted at 20 °C throughout the measurement unless otherwise specified.

### 2.11 Protein profile of MFG and oleosomes

Protein profile of the samples was characterised by sodium dodecyl sulphate poly acryl amid gel (SDS-PAGE) according to the method described by De Chirico et al. (2018) with a few modifications. Firstly, dried samples were diluted in 1 mL of 2 (g/dL) SDS solution to denature the proteins. Then, 20 μL of samples were mixed with a certain amount of reducing (Laemmli) buffer (20 μL for oleosomes and 15 μL for MFGs) and then heated for 5 min at 95 °C. Proteins were resolved by SDS-PAGE using 4-20% polyacrylamide gels (Criterion TGX Stain-Free precast gels, Bio-Rad, USA). SDS gels were positioned in a SE600 Bio-Rad separation unit and suspended in running buffer (25 mM Tris, 192 mM glycine, and 0.1% SDS; pH 8.3). The conditions for electrophoresis were 200 V voltage and 45 min run duration for oleosomes and 150 V voltage and 50 min run duration for MFGs. After the electrophoresis process, gels were imaged using Bio-Rad Gel DOC XR system with the gel activation time of 30 s.

### 2.12 Statistical analysis

All the experiments were repeated (at least) in triplicate. A one-way analysis of variance (ANOVA) using SPSS software version 26 (SPSS software, V26, SPSS Inc., Chicago, USA) was used to study data. The significant difference among samples were assessed based on a 95% confidence limit using Duncan's test. All the graphs were plotted by Origin Pro 2022b software (OriginLab, MA, USA).

## 3 Results and discussions

### 3.1 Lipase activity and microbial count of the heat-treated oleosomes

Lipase and microbial activity would lead to the release of free fatty acids from the natural lipid droplets affecting their surface tension behaviour. It is important to deactivate lipase and microorganisms to minimise their activity. To achieve deactivation 15 (g/dL, lipid based) emulsions were thermally treated at 75 °C for 5 min. Data compiled in Table 1 show the lipase activity and the total aerobic colony count (ACC) of the oleosome samples. Lipase activity became negligible after thermal treatment and ACC decreased significantly (10 times), limiting the impact of microbial metabolic activity within the timescale of these experiments.

**Table 1:** Lipase enzyme activity and ACC of non- and heat treated oleosomes (75 °C for 5 min).

Sample	Lipase activity (nkat/mg protein)	ACC (LogCFU/mL)
Non-heat treated oleosomes	2.00^A^ ± 1.20	10.13^a^ ± 0.15
Heat treated oleosomes	0.02^B^ ± 0.00	0.98^b^ ± 0.83

∗ Values are reported as average ± STD. Values with different letters in each column show significant differences (*p < 0.05*).

### 3.2 Homogenisation efficiency and interfacial adsorption of oleosomes as affected by purification

Table 2 shows the efficiency of homogenisation at 207 MPa, 2x on the size reduction of various oleosomes based on their purity. COB emulsions showed the highest degree of change (around 85%) after homogenisation while the impact of homogenisation at the mentioned pressure/pass was negligible as the oleosomes were purified (i.e., WOB emulsion). This shows that high pressure homogenisation at 207 MPa, 2x could not reduce the size (break the oleosomes droplets) when they are being purified. This might be because of the small size of the WOB emulsion droplets which might not collide with each other effectively in the interaction chamber of the homogeniser.

**Table 2:** Average droplet diameter, surface tension (ST), and equilibrium time (Eq) of crude oleosomes (COB) and washed oleosomes (WOB) before and after homogenisation at 207 MPa, 2 passes (2x). Both emulsions were fixed at 15 g/dL lipid-based concentration for homogenisation and diluted to 0.1 (g/dL) for surface tension measurements.

	COB	WOB
D_4,3_ before homogenisation (μm)	2.08 ± 0.97	1.04 ± 0.30
D_4,3_ after homogenisation (μm)	0.30 ± 0.15	0.98 ± 0.11
Eq. ST before homogenisation (mN/m)	30.31 ± 0.34	33.01 ± 0.20
Eq. ST after homogenisation (mN/m)	44.98 ± 0.40	33.23 ± 0.65
Eq. time before homogenisation (minutes)	145.00 ± 3.00	102.00 ± 10.00
Eq. time after homogenisation (minutes)	203.00 ± 5.73	105 ± 5.15

Table 2 also shows the interfacial properties of oleosomes before and after homogenisation. The equilibrium surface tension was significantly higher in the homogenised COB compared to native COB and the equilibrium time changed from 145 min to 203 min showing that there was a change in the properties of the interfacial-active compounds which delayed the interface saturation time after homogenisation; while there was no significant changes in the equilibrium time of the purified oleosomes (WOB) which show possible similar structure of adsorbed compounds at the interface. Therefore, we selected COB emulsion to carry out the rest of the experiments to understand the mechanism of homogenisation on oleosomes better and to understand which mechanism(s) are possibly responsible for the alteration in the surface adsorption of them.

### 3.3 Homogenisation of milk fat globules and oleosomes

Lipid droplet size affects how well fat-based structures like MFGs and oleosomes perform during food processing and in the final product. We aimed to understand how increasing lipid concentration impacts the ability of a high-pressure processing equipment, i.e., microfluidizer, to effectively reduce droplet size. This would help to determine if there is a limit beyond which the process becomes ineffective. To test this, we processed MFGs and oleosomes at different phase volumes. Homogenisation was carried out at 207 MPa with double passes.

The D4,3 values for all samples are presented in Table 3. A reduction in the oleosome size compared to the control was observed upon high-pressure processing. However, an increase in the oleosome phase volume did not lead to a significant decrease in D4,3 values (applied pressure of 207 MPa with 2 passes). This suggests that within the tested concentration range (i.e., 1-25 g/dL lipid-based), the microfluidizer effectively reduced oleosome size to submicron levels. The size reduction shows there was a sufficient amount of surface-active materials (i.e., phospholipids and/or proteins) available which could stabilise the newly formed oleosome droplets. However, it is important to consider that beyond a concentration of 25 (g/dL), the efficiency of homogenisation at the applied pressure might diminish, potentially due to re-coalescence resulting from insufficient amount of adsorbed surface-active materials (McClements, 2004; Salum et al., 2024).

**Table 3:** Size reduction and total surface area of milk fat globule (MFG) and oleosomes at different lipid-based concentrations after passing through a microfluidizer at pressure of 207 MPa after 2 passes. Control refers to non-homogenised samples.

Sample	Concentration (g/dL)	D4,3 (μm)	Total surface area (m^2^) per m^3^ of emulsion
MFG	Non-homogenised	4.12^a^ ± 1.32	1.46 × 10^6^
	1	1.76^b^ ± 0.81	3.41 × 10^6^
	5	0.80^c^ ± 0.53	7.50 × 10^6^
	15	0.83^c^ ± 0.34	7.23 × 10^6^
	20	2.27^b^ ± 0.86	2.64 × 10^6^
	25	3.76^ab^ ± 0.60	1.60 × 10^6^
Oleosome	Non-homogenised	2.08^d^ ± 0.97	2.90 × 10^6^
	1	0.41^e^ ± 0.31	14.63 × 10^6^
	5	0.38^e^ ± 0.20	15.79 × 10^6^
	15	0.30^e^ ± 0.15	20.00 × 10^6^
	20	0.28^e^ ± 0.11	21.43 × 10^6^
	25	0.34^e^ ± 0.72	17.65 × 10^6^

**∗** Values are reported as average ± STD. ∗∗ Values with different symbols show a significant difference (*p < 0.05*).

In contrast, for MFGs a substantial decrease in fat globule size was observed up to a concentration of 15 g/dL. Beyond this point, homogenisation became progressively less effective, as seen with the 20 and 25 g/dL samples. Specifically, the average droplet diameter of MFGs was reduced by almost 80% from the control to the sample with a concentration of 15 g/dL, while the reduction was comparatively lower at around 45% and 9% for concentrations of 20 and 25 g/dL, respectively. This reduction trend in homogenisation efficiency suggests that at higher MFG concentrations factors such as viscosity-induced resistance to shear forces could hinder the re-emulsification process (Innocente et al., 2009). In addition, the observed reduction trend could be due to the re-coalescence after homogenisation. In other words, the higher the phase volume, the higher the number of droplets and therefore the higher the number of droplets collisions leading to coalescence and larger droplet size (Floury et al., 2000; Walstra & van Vliet, 2003, pp. 9–30).

Given the observed impact of MFG concentration on the microfluidizer's re-emulsification efficiency, and to maintain consistency between MFGs and oleosomes, a lipid-based concentration of 15 g/dL was selected for the rest of the experiments. This dispersed (lipid) phase concentration ensures effective droplet size reduction while avoiding potential limitations associated with higher lipid phase concentrations.

Data compiled in Table 3 also show the total surface area of emulsion droplets for the non-homogenised (control) and homogenised samples for both MFG and oleosomes. Surface area increased significantly upon homogenising MFGs. The 5 (g/dL) sample had the highest total cumulative surface area (almost 5-fold to the control sample). The increase in the surface area decreased afterwards (i.e., from 15% onwards) with the only around 1.05 times to the control. In oleosomes, on the other hand, the total surface area increased by 5 times in the 15 g/dL sample compared to the control and did not change significantly thereafter. These data along with the size reduction data (D_4,3_ values), show an increase in the emulsification capacity of MFG and oleosomes upon homogenisation.

**Fig. 1:**
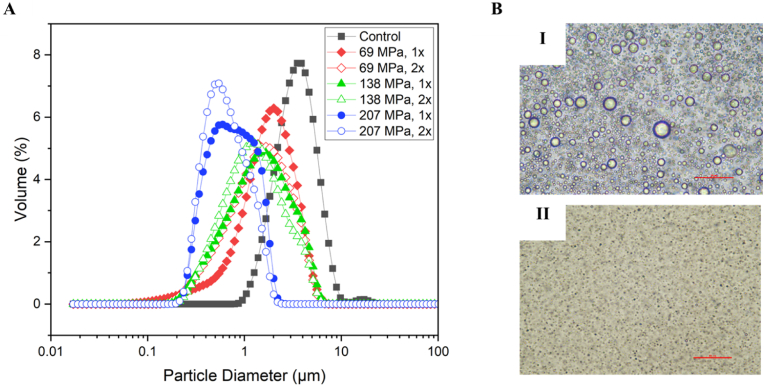
A) Particle size distribution of milk fat globules (MFGs) as a function of homogenisation pressure. B) Bright field micrographs of (i) non-homogenised and (ii) homogenised (207 MPa, two passes - 2x) MFG. Scale bars are 50 μm. Full and empty symbol in panel A represent one or two passes respectively, while diamond, triangle and circle represent 69, 138, and 207 MPa, respectively.

The impact of number of passes on the final particle size distribution (PSD) of MFGs was investigated and the respective PSD and micrographs are shown in Fig. 1A & B, respectively. The native MFG (control) had the largest distribution peak with a shift in the centre value of distribution to the lower particle diameters after homogenisation. The centre peak in the control sample was around 4 μm with another small peak around 18 μm. Following homogenisation, centre peaks were shifted to smaller values (for example, around 3 μm and 0.4 μm for 69 MPa 1x and 207 MPa 2x, respectively). As shown in Fig. 1A, treating the MFG dispersion at 207 MPa after two passes (2x) resulted in the smallest distribution peak among the other treatments. The observed results from the particle size distribution graph have been confirmed by the micrographs of the samples (Fig. 1B): droplets appear significantly smaller after homogenisation (207 MPa, 2x) compared to control sample (i.e. native MFG). Micrographs also suggest that after homogenisation no emulsion instability (e.g., partial and/or complete coalescence) had occurred. Barnadas-Rodríguez and Sabés (2001) reported that mean size of the liposomes decreased significantly with an increase in the pressure and number of passes in the microfluidizer indicating a direct correlation between the size distribution of droplets and the applied pressure as also being observed in our findings.

In addition, when operating at a given pressure, performing an additional pass resulted in a more uniform size distribution; suggesting that the second pass through the microfluidizer helped the distribution peaks to be more of narrower distributions. This can be attributed to the longer residence time of the lipid droplets in the interaction chamber which results in an increased homogeneity (Dumay et al., 2013).

**Fig. 2:**
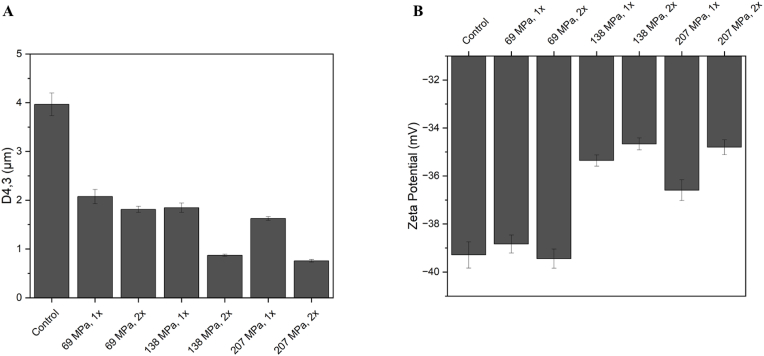
Average diameter (A) and zeta potential (B) of MFG as a function of homogenisation pressure and number of passes.

The average droplet diameter (D4,3) of the non-homogenised and homogenised MFGs at the tested pressures are shown in Fig. 2A. A significant size reduction was observed at all applied pressures. When applying 69, 138, and 207 MPa a 48.3%, 53.9% and 59.5% reduction was achieved compared to the control. Furthermore, by applying a second pass a reduction of 54.8%, 78.3%, and 81.1% were obtained at 69, 138, and 207 MPa, respectively (compared to the control).

One probable reason for the difference in size reduction in more passes could be due to the compositional changes in the membrane after the homogenisation (re-emulsification). As Thompson and Singh (2006) reported, the phospholipid content change could affect the sensitivity of emulsion droplets to the breakage during the homogenisation. They reported that samples with higher phospholipid concentrations had less sensitivity to the shear and turbulence, and droplets could resist the deformation more than samples with less phospholipids (i.e., adsorbed phospholipids at the oil-water interface). Bachmann et al. (1993) reported that droplet size reduction in a high-pressure homogenizer was less effective when the phospholipid content is more than 10%. The triple layer of native MFGs membrane consist of many phospholipid types which are densely packed around the oil core giving MFG membrane with higher PL content, more resistance to the shear forces during the homogenisation (Lu et al., 2021; Sánchez-Juanes et al., 2009).

Surface charge of the MFG suspensions before (control) and after homogenisation at different pressures and passes are shown in Fig. 2B. Zeta potential of the native MFG (control, non-homogenised sample) was measured as −39.2 mV. This value was in line with previous reports such as Sun et al. (2022) at the same pH value; however, there are some studies reported lower zeta potential values for MFGs at near- and neutral pH (6-7) such as Lu et al. (2021) and Michalski et al. (2002). The differences in the surface charge of MFGs in different studies could be attributed to the differences in the primary source of MFGs (and different membrane compositions) as described by Sun et al. (2022). Following homogenisation, surface charge of MFG suspensions decreased. The reduction was less obvious at low pressures, while reduction in the surface charge was observed more after treating samples at 138 and 207 MPa. This phenomenon can be related to the loss of charged fragments in MFGM when high pressures applied during the homogenisation (i.e., part of the membrane molecules may be mechanically removed from the interface and disperse in the serum phase). It has been reported that some of the fragments in MFGM are responsible for MFG's native surface charge. Among them, phospholipids and glycoproteins are the most abundant components which give MFGs a negative charge (Obeid et al., 2019). The MFGM compositional changes during homogenisation (e.g., loss of phospholipids) could also affect the total charge of MFG's surface.

**Fig. 3:**
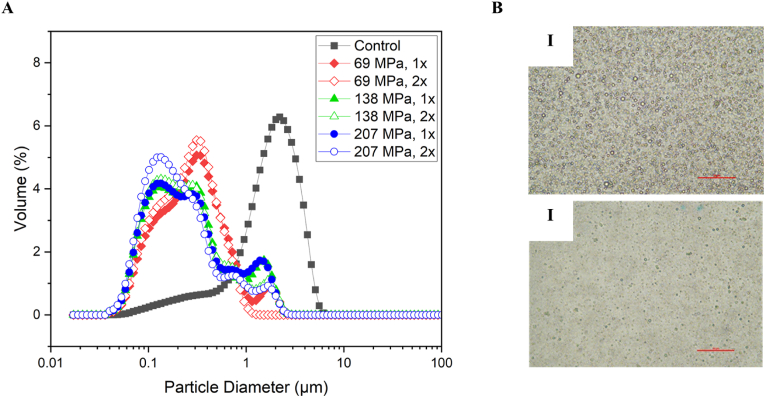
A) Particle size distribution of oleosomes as a function of homogenisation pressure. B) Bright field micrographs of (i) non-homogenised and (ii) homogenised (207 MPa, two passes - 2x) oleosomes. Scale bars are 50 μm. Full and empty symbol in panel A represent one or two passes respectively, while diamond, triangle and circle represent 69, 138, and 207 MPa, respectively.

Size distribution of native and homogenised oleosomes and their respective micrographs are shown in Fig. 3A and B, respectively. The lowest homogenisation regime (i.e., 10k and 1x) shifted the distribution peak to the smaller size value region significantly (compared to native) while any additional pressures and/or number of passes did not change the distribution peaks further (compared to 10k and 1x). As shown in Fig. 3A, neither of the distribution peaks in oleosome samples were monomodal. In control, the centre peak was around 4 μm with at least one shoulder peak below 1 μm. After homogenisation at 10k 1x, the centre peak shifted to around 0.4 μm with a shoulder peak around 0.1 μm and another small peak at around 2 μm. This shift continued till the highest applied pressure (207 MPa) 2x, in which two small peaks were observed at around 2 and 1 μm and the main centre peak shifted to 0.2 μm. These observations could be because of the size of oleosomes which are much smaller compared to MFGs. The small lipid droplets might not efficiently collide with each other in the interaction chamber an as a result, despite the breakage of the majority of droplets, some of them leave the homogeniser as intact droplets (small peaks around oleosomes natural size). Micrographs (Fig. 3B) confirm the size reduction of oleosomes during the homogenisation and similar to MFGs, there was no sign of emulsion instability after homogenisation.

**Fig. 4:**
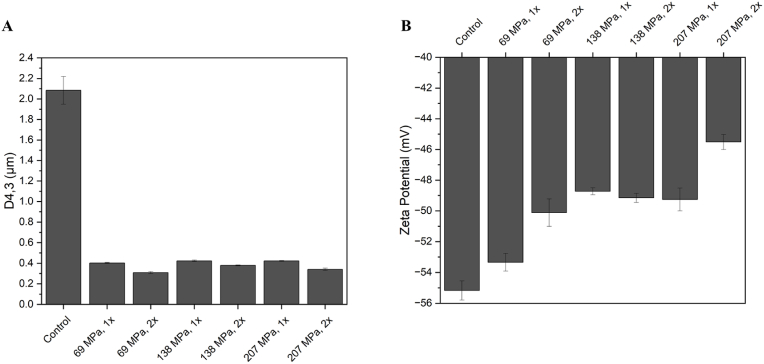
Average droplet diameter (A) and zeta potential (B) of oleosomes as a function of homogenisation pressure.

Fig. 4A shows the average droplet diameter of native and homogenised oleosomes. D4,3 was recorded as 2.1 μm for the non-homogenised sample (control) which is close to the native size of sunflower oleosomes that reported in the literature (Aliyari et al., 2025; Karefyllakis et al., 2019) and it decreased by 81% to 0.4 μm after homogenising the sample at 69 MPa, 1 pass. Any further pressure and/or number of passes did not change the D4,3 significantly. These observations suggest that, unlike MFGs, increased residence time of oleosomes in the interaction chamber (because of multiple passes through the microfluidizer) had no obvious impacts on their breakdown and size reduction. As Yang et al. (2025) described in their work, oleosins in oleosomes’ membrane penetrates deeply into the core TAGs and make a solid-like viscoelastic film at the interface. As a result of oleosomes first breakage during the homogenisation and re-adsorption of oleosins to the oil-water interface, the newly formed oleosin dominant membrane can resist the applied pressure and simply just reform rather than more breakages and size reductions.

Surface charge of oleosomes was affected by the homogenisation (as shown in Fig. 4B). Like MFGs, homogenisation caused a reduction in the surface charge of oleosomes compared with the control and the lowest zeta potential value was recorded for the highest homogenisation pressure (30k) after two passes (2x). Zeta potential was decreased from −55 mV in control to −45 mV in homogenised sample at 207 MPa, 2 passes. However, the lowest recorded zeta potential was still in the stable region of the emulsions (Aliyari et al., 2025); means that the electrostatic repulsion was still sufficiently high to provide electrostatic stability to oleosomes. The charge reduction of oleosomes after homogenisation might be due to the structural changes of them. The applied pressure through homogenisation might have changed the composition of oleosomes’ membrane. As discussed in a previous study, the main molecules within the membrane structure responsible for the net charge of oleosomes are phospholipids and integral proteins (mainly oleosins) (Nikiforidis, 2019). Any possible changes in the composition of oleosome membrane will lead to a change in the charge as we have captured in this study. To understand the changes in the membrane after homogenisation, further tests have been done, and they will be discussed in the following sections.

### 3.4 Impact of homogenisation on the protein profile of oleosomes and milk fat globules

**Fig. 5:**
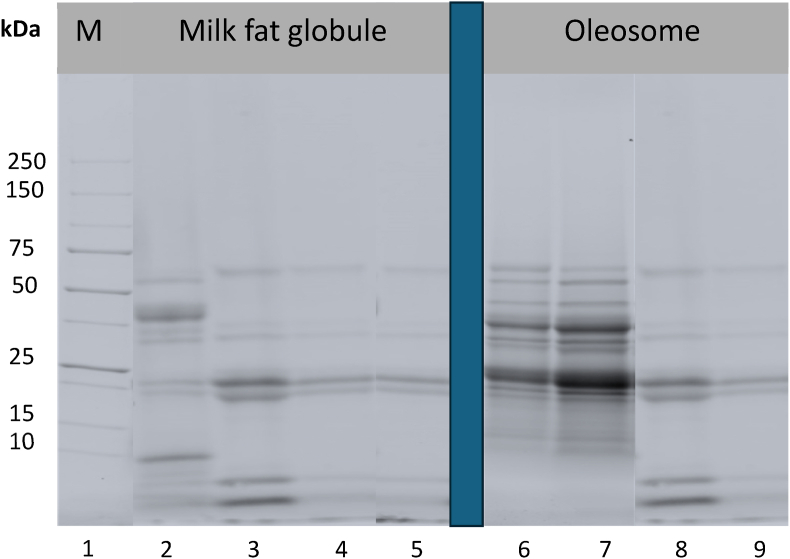
Protein profile of native and homogenised (207 MPa, 2x) milk fat globule and oleosome creams and their serum phases assessed via SDS-PAGE assay. Lane 1: Marker (protein standard); lane 2: Native MFG cream; lane 3: Homogenised MFG cream; lane 4: Native MFG serum; lane 5: Homogenised MFG serum; lane 6: Native oleosome cream; lane 7: Homogenised oleosome cream; lane 8: Native oleosome serum; Lane 9: Homogenised oleosome serum.

Data shown in Fig. 5 is the SDS-PAGE assay to characterise the protein profile of both native and homogenised creams and serums for oleosomes and milk fat globules (MFGs). The primary proteins identified in native MFG cream were mucin I and butyrophilin, both of which are key structural components of the milk fat globule membrane (MFGM) (Cebo et al., 2010). However, following homogenisation, casein fractions became more predominant within the membrane, suggesting substantial adsorption of plasma-phase caseins onto the newly formed fat globules (Obeid et al., 2019).

Additionally, the presence of native membrane proteins originally associated with the native MFGM was more pronounced in the homogenised serum phase. This indicates that during homogenisation, caseins (initially dispersed in the plasma phase surrounding MFGs) adsorb onto the surface of newly formed fat droplets, effectively stabilizing them. Concurrently, native MFGM proteins, including mucin I and butyrophilin, are displaced and released into the plasma phase (Lee & Sherbon, 2002). This phenomenon aligns with previous research, which has demonstrated the disruption and reorganisation of the MFGM structure during high-pressure homogenisation.

For oleosomes, characteristic proteins (namely oleosins (∼20 kDa), caleosins (∼35 kDa), and steroleosins (∼50 kDa)) were clearly identifiable in the native cream (Fig. 5). This protein profile is consistent with previous studies, which have highlighted the role of oleosins in stabilizing the lipid core of oleosomes and maintaining their structural integrity (Tzen & Huang, 1992). The preservation of these proteins post-homogenisation suggests that, unlike MFGs, oleosomes retain their protein-stabilised interface even after mechanical processing, potentially due to the robustness of their intrinsic phospholipid-protein membrane. In addition, it has been reported that oleosins (the main interfacial protein of oleosomes) penetrate deep into the oleosomes’ main TAG core; as a result, protecting the membrane against destabilisation (Yang et al., 2025; Şen et al., 2024).

### 3.5 Impact of shear homogenisation on the surface adsorption activity of milk fat globules and oleosomes

#### 3.5.1 Milk fat globules behaviour at the air-water interface

**Fig. 6:**
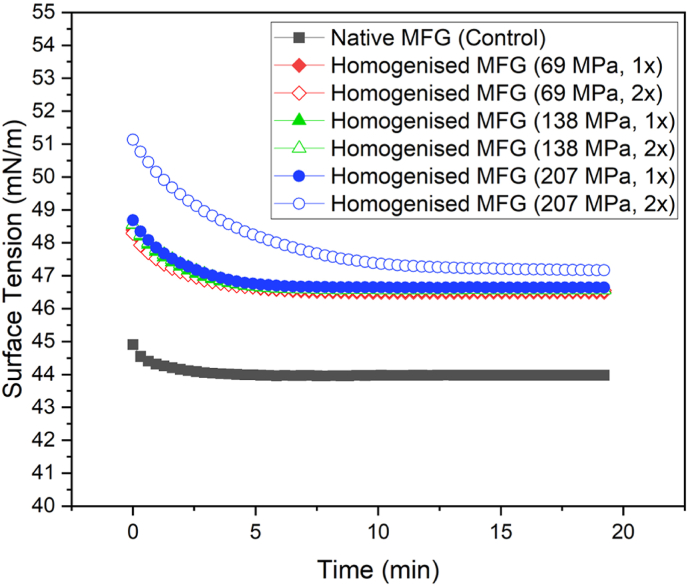
Surface adsorption behaviour of MFGs as a function of homogenisation pressure (the concentration of all the samples was 0.1 g/dL). All the analysis was carried out at 20 °C. Full and empty symbol represent one or two passes respectively, while diamond, triangle and circle represent 69, 138, and 207 MPa, respectively.

The surface tension behaviour of milk fat globules (MFGs), both in their native (control) and homogenised states (pressures of 69, 138, and 207 MPa with one and double passes), at a concentration of 0.1 g/dL is shown in Fig. 6. In the native MFG sample, surface tension exhibited a rapid decline, followed by reaching the equilibrium state within approximately 3 min. The final surface tension value at equilibrium for the control sample was recorded at 44.00 ± 1.13 mN/m. Homogenisation of MFGs led to two distinct changes in their adsorption behaviour at the air-water interface compared to the non-homogenised MFGs:

I) A more gradual decrease in surface tension.
II) A longer time required to reach equilibrium, indicating delayed saturation of the air-water interface by lipid droplets.

As illustrated in Fig. 6, the extent of homogenisation, dictated by applied pressure and the number of passes through the microfluidizer, significantly influenced the surface tension trend. The highest equilibrium surface tension value (47 mN/m) was observed in samples subjected to homogenisation at 207 MPa for two passes (2 × ) through the microfluidizer. Moreover, the equilibrium time for these samples was notably extended to 12 min, in contrast to the 3-min equilibrium observed for the control sample. One probable explanation for these observations is the structural alteration of the MFG membrane (MFGM) during the homogenisation (within the interaction chamber of the microfluidizer). The MFGM, a complex biological membrane composed of proteins, phospholipids, and other amphiphilic molecules, plays a critical role in the surface activity of MFGs (Phipps & Temple, 1982). During homogenisation, high pressure disruption causes fragmentation and rearrangement of the MFGM, leading to altered interfacial properties (Gassi et al., 2008). This modification affects the ability of lipid droplets to adsorb at the air-water interface, thereby influencing both the rate of surface absorption with surface tension reduction and the equilibrium value achieved.

Studies have demonstrated that the effectiveness of MFG adsorption at the interface depends on the availability and conformation of interfacial components, such as membrane-associated proteins and phospholipids (Lopez, 2011). Homogenisation-induced replacement of native phospholipids with proteins such as caseins and whey proteins around the main core reduces the interfacial activity of the droplets, resulting in higher equilibrium surface tension values and prolonged adsorption times (Lee & Sherbon, 2002).

Additionally, the increase in the equilibrium surface tension values with higher homogenisation pressures may be attributed to the formation of smaller droplets with reduced interfacial coverage by membrane components. As reported by Schultz et al. (2004) smaller droplets generated through homogenisation possess higher Laplace pressures, which influence interfacial dynamics. The reduction in available native surfactant molecules post-homogenisation leads to a slower surface tension decrease and increased equilibrium values, as observed in our study. The observed correlation between homogenisation parameters (pressure and number of passes) and surface tension trends suggests that more intense homogenisation disrupts MFGM integrity more significantly, altering the balance of interfacial-active components. The extended equilibrium time observed in the homogenised samples aligns with prior findings where homogenisation caused restructuring of the interfacial layer, requiring additional time for droplet adsorption (Bourlieu et al., 2012).

Another probable reason for this behaviour is the effect of homogenisation in creating extra available surface. In another words, because of homogenisation, droplets broke down and their size decreased (as discussed in section 3.2). This size reduction of fat globules led to an increase in their surface area (as shown in Table 3). The increased surface area of newly formed droplets can be stabilised by free small molecular weight surfactants (emulsifiers) and because of that, fewer of them are available to freely move towards the interface; thus, the equilibrium surface tension of the homogenised samples was higher than the control.

#### 3.5.2 Oleosomes behaviour at the air-water interface

**Fig. 7:**
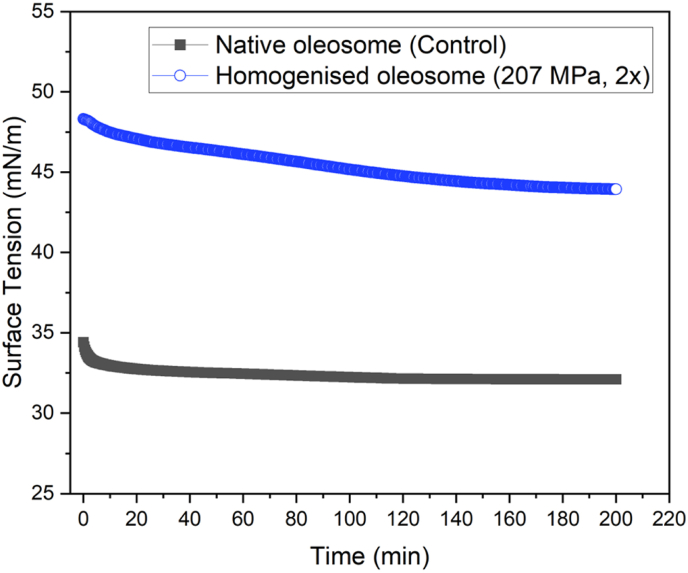
Surface adsorption behaviour of non-homogenised (black square; control) and homogenised oleosomes (207 MPa - 2x; red circles). Concentration of all the samples were fixed at 0.1 g/dL. All the analysis was carried out at 20 °C.

The surface tension response of oleosomes as a function of homogenisation at a concentration of 0.1 g/dL is shown in Fig. 7. Due to the overlap in surface tension trends across different homogenisation regimes, only two representative plots are shown: the non-homogenised oleosomes (control) and the homogenised oleosomes processed at 207 MPa for two passes (2 × ) through the microfluidizer. As observed for MFGs (Fig. 6), the control oleosome sample exhibited a rapid decline in surface tension within the first few minutes, followed by a stabilisation phase where equilibrium was achieved. However, homogenisation resulted in significant changes in the interfacial behaviour of oleosomes, as indicated by two key observations: I. an extended equilibrium time of 160 min, and II. a significantly higher equilibrium surface tension of 45.00 ± 0.80 mN/m, compared to the control sample. As mentioned in section 1, the specific structure of the oleosomes provides them with a stable interfacial structure, contributing to their efficient adsorption and rapid equilibrium at the air-water interface in their non-homogenised state. Upon homogenisation, high-pressure disruption alters the integrity of the oleosome structure, affecting the adsorption kinetics and equilibrium surface tension. The observed increase in equilibrium time and surface tension values suggest that homogenisation results in significant structural modifications. The initial breakage of oleosome droplets increased the surface area. The rapid adsorption of oleosins to the newly formed surfaces followed by the carryover of phospholipids in the serum phase, caused a delay in the adsorption of homogenised droplets to the air-water interface and higher final surface tension compared to the control (non-homogenised) (Ho et al., 2022; Lin et al., 2025).

### 3.6 Impact of the temperature on the surface activity of milk fat globules and oleosomes

**Fig. 8:**
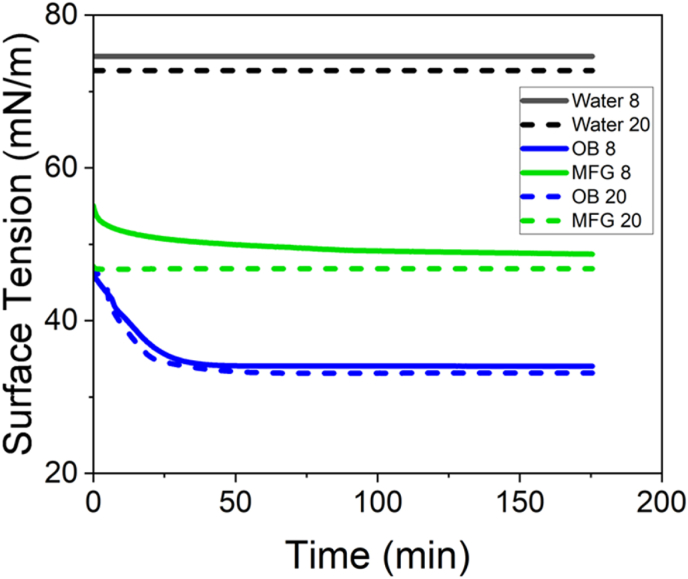
Surface adsorption behaviour of native milk fat globules (MFG) and oleosomes (OB) as a function of temperature (concentrations for all samples were fixed at 0.1 g/dL). Dashed and continuous lines represent measurements taken at 20 and 8 °C, respectively. The value of surface tension for water is indicated in dark grey, while MFG and OB are in green and blue, respectively.

The impact of temperature (8 °C and 20 °C) on the surface tension activity of milk fat globules (MFGs) and oleosomes (OB) is depicted in Fig. 8. A low temperature (8 °C) was selected to be sufficiently different from ambient conditions, allowing for observable differences in the adsorption behaviour, while also remaining distinct from the maximum density point of water (4 °C). Water density increases with decreasing temperature until it reaches a maximum at 4 °C (1.00 g/mL in pure water). Below this temperature, the density decreases due to the expansion of the water network, forming ice-like clusters, which disrupt the compact hydrogen-bonding structure and increase volume (Cho et al., 1996).

The surface tension of MFGs was more significantly affected by temperature changes than that of oleosomes. At equilibrium, the surface tension of MFGs at 8 °C was higher than at 20 °C. A similar trend was observed for oleosomes, but the magnitude of change was much smaller. This difference can be attributed to the variation in the solid fat content (SFC) of the lipid phase at different temperatures. At 8 °C, more fat is crystallised than 20° in MFGs (higher SFC at 8 °C than 20 °C). The greater sensitivity of MFGs to temperature changes can be explained by their lipid composition as analysed by GC-MS and shown in Table 4. Milk fat is rich in saturated fatty acids, such as palmitic acid (C16), which exhibit significant changes in SFC with temperature variations. At lower temperatures, a higher proportion of these lipids remain in the solid phase, increasing the rigidity of MFGs and altering their interfacial properties (Lopez, 2011). In contrast, the studied oleosomes, which are primarily composed of liquid oil as the main core, as it contains a higher proportion of unsaturated fatty acids, such as linoleic acid (C18). These lipids remain predominantly in the liquid state across the tested temperature range, resulting in minimal SFC-related structural changes and a relatively stable interfacial behaviour.

**Table 4:** Fatty acid profile of sunflower oil and milk fat using the GC-MS.

Fatty acid	Sunflower oil	Milk fat
Short-chain FAs (<C12)	N.D.	∼0.95
Lauric acid	N.D.	2.01 ± 0.12
Myristic acid	0.10 ± 0.01	6.67 ± 1.51
Myristoleic acid	N.D.	0.05 ± 0.00
Palmitic acid	5.75 ± 1.04	40.87 ± 0.60
Palmitoleic acid	0.03 ± 0.00	1.45 ± 0.04
Heptadecanoic acid	N.D.	0.46 ± 0.21
Stearic acid	2.20 ± 1.03	19.94 ± 1.87
Elaidic acid	N.D.	N.D.
Oleic acid	40. 76 ± 1.98	21.09 ± 2.04
Linolelaidic acid	0.12 ± 0.00	0.02 ± 0.00
Linoleic acid	50.03 ± 2.98	1.98 ± 0.31
Arachidic acid	0.04 ± 0.00	0.17 ± 0.00
3n6 Linolenic acid	N.D.	0.31 ± 0.06

N.D. means not detected. ∗ Values are reported as average ± STD.

Additionally, the higher SFC in MFGs at 8 °C may reduce the mobility of interfacial components, slowing the adsorption kinetics and leading to higher equilibrium surface tension. This effect is less pronounced in oleosomes, as their monolayer-stabilised structure remains fluid and dynamic across the tested temperatures, allowing for efficient interface formation despite the cooling effect.

### 3.7 Stability of oleosomes upon surface adsorption (a comparison between non-homogenised and homogenised oleosomes)

**Fig. 9:**
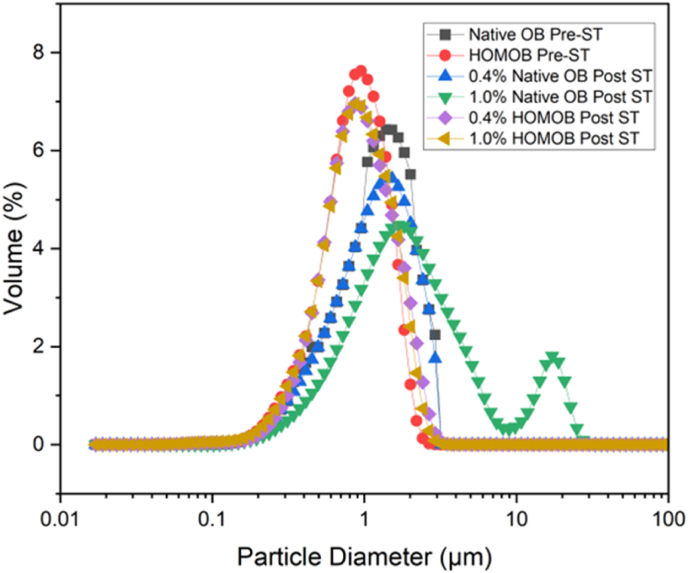
Particle size distribution of different concentrations of native and homogenised oleosomes (0.4 and 1.0, g/dL) upon surface saturation.

Size distribution of native and homogenised oleosomes before and after occupying the air-water interface is shown in Fig. 9. Prior to the surface tension measurements (pre-saturation), lipid droplets were distributed as mono modal peak. However, at high concentrations (i.e., 1.0 g/dL), a second peak appeared around 20 μm which was not observed in any other samples. This second peak in the large size region might explain the possible destabilisation of native oleosomes upon adsorbing to the air-water interface. This, however, was not the case for the homogenised oleosomes. Even at high concentrations (1.0, g/dL), there was no change in the droplets’ distribution peaks before and after (post-saturation) surface adsorption which implies that homogenised oleosomes remained intact after reaching the equilibrium at the air-water interface and no destabilisation mechanism occurred.

**Fig. 10:**
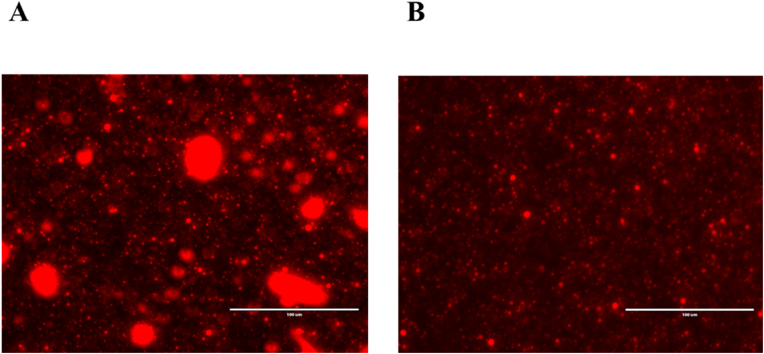
Fluorescence images of intact (A) and homogenised (B) oleosomes upon reaching to the equilibrium state. Scale bars are 100 μm.

Nile red was used to observe the morphological changes of the native and homogenised oleosome droplets at the interface. Fluorescence micrographs (Fig. 10) confirm that there were significantly larger lipid droplets in the native oleosome sample after reaching the equilibrium while in homogenised oleosomes, despite the significantly longer equilibrium time compared to the control, droplets seem to remain intact upon surface adsorption. The large red regions in the native sample show that destabilisation led to fat partial and/or complete coalescence. As explained in the previous sections, homogenisation of oleosomes led to changed membrane composition which made the membrane more rigid, and stable. To understand the interfacial components at the air-water interface, using RH-DOPE for phospholipids and Nile Blue for proteins could provide more compositional information in a future study.

## 4 Conclusions

This study showed that high-pressure and high-shear homogenisation (microfluidization) could alter the composition of native dairy and plant-based lipid droplets. The association of casein fractions to the newly formed MFGs changes their surface properties significantly, for example the time to reach equilibrium surface tension the air-water interface. The degree of change was in correlation with the intensity of homogenisation. A similar behaviour was observed in oleosomes where the surface adsorption of lipid droplets was affected by high-pressure homogenisation. Protein analysis of the native and homogenised creams and serums confirmed the significant changes in the membrane composition of both MFG and oleosomes after microfluidization. The observed impact of homogenisation on MFG and oleosome surface tension behaviour underscores the importance of optimising processing conditions to maintain desired interfacial properties. A deeper understanding of these mechanisms can inform the development of plant-based emulsions with tailored functional properties for applications in dairy alternatives, cosmetics, and pharmaceuticals. This study signifies the importance of optimising the processing conditions to gain tailored lipid droplets for applications requiring controlled surface activity, such as in the development of foams and emulsified dairy products.

## CRediT authorship contribution statement

**Amin Aliyari:** Writing – original draft, Visualization, Validation, Software, Resources, Methodology, Investigation, Formal analysis, Data curation, Conceptualization. **Vincenzo di Bari:** Writing – review & editing, Supervision, Investigation. **Liam P.D. Ratcliffe:** Writing – review & editing, Supervision. **Yuanzhang Dong:** Writing – review & editing. **David Gray:** Writing – review & editing, Supervision, Project administration, Investigation, Funding acquisition, Conceptualization.

## Declaration of competing interest

The authors declare that they have no known competing financial interests or personal relationships that could have appeared to influence the work reported in this paper.

## Data Availability

Data will be made available on request.
